# Microbiological Quality of Coconut Water Sold in the Grande Vitória Region, Brazil, and Phenogenotypic Antimicrobial Resistance of Associated Enterobacteria

**DOI:** 10.3390/microorganisms12091883

**Published:** 2024-09-12

**Authors:** Valéria Modolo Peterle, Juliana Aliprandi Bittencourt Cardoso, Carolina Magri Ferraz, Delcimara Ferreira de Sousa, Natália Pereira, Alessandra Figueiredo de Castro Nassar, Vanessa Castro, Luis Antonio Mathias, Marita Vedovelli Cardozo, Gabriel Augusto Marques Rossi

**Affiliations:** 1 Department of Veterinary Medicine, University of Vila Velha (UVV), Vila Velha 29102-920, ES, Brazil; 2 Department of Pathology, Reproduction and One Health, Sao Paulo State University (UNESP), Jaboticabal 14884-900, SP, Brazil; 3 Instituto Biológico (IB) de São Paulo, Rua Conselheiro Rodrigues Alves, 1252, São Paulo 04014-002, SP, Brazil

**Keywords:** antibiotics, *bla*_CTX-M-2_, *Citrobacter*, *Enterobacter*, *Enterobacteriaceae*, ESBL, food microbiology, *Klebsiella*, *Kluyvera*

## Abstract

This study aimed to evaluate the microbiological quality of coconut water sold from street carts equipped with cooling coils or refrigerated at bakeries in the Grande Vitória Region, Brazil. Additionally, it assessed the phenotypic and genotypic antimicrobial resistance profiles of isolated enterobacteria. The results indicated that coconut water sold at street carts had lower microbiological quality compared to refrigerated samples, as evidenced by significantly higher counts of mesophilic microorganisms. Using MALDI-TOF, the following opportunistic pathogens were identified: *Citrobacter freundii*, *Enterobacter bugandensis*, *E. kobei*, *E. roggenkampii*, *Klebsiella pneumoniae*, and *Kluyvera ascorbata*. Three isolates—*E. bugandensis*, *K. pneumoniae*, and *K. ascorbata*—were classified as multidrug-resistant (MDR). Widespread resistance to β-lactams and cephalosporins was detected, and some isolates were resistant to quinolones, nitrofurans, and phosphonic acids. The gene *bla*_CTX-M-2_ was detected in *C. freundii*, *E. bugandensis*, *E. kobei*, and *K. ascorbata*. However, genes *bla*_NDM_, *bla*_KPC_, *bla*_CMY-1_, and *bla*_CMY-2_ were not detected in any isolate. The findings underscore the need to enhance good manufacturing practices in this sector to control the spread of antimicrobial resistance (AMR). To our knowledge, this is the first study documenting the presence of potentially pathogenic enterobacteria in coconut water samples and their associated phenotypic and genotypic AMR profiles.

## 1. Introduction

The growing global popularity of coconut water is attributed to its favorable nutritional profile. This refreshing, low-calorie beverage contains 5–9% total soluble solids, which include soluble sugars, minerals, amino acids, enzymes, organic acids, fatty acids, vitamins, and small amounts of phenolic compounds [1,2].

In Brazil, coconuts water is available in several forms: (i) industrially sterilized products, which have a long shelf life at room temperature and are commonly found in supermarkets; (ii) “natural” versions that are produced by small food companies and typically sold in bakeries or supermarkets and are kept refrigerated and have a shelf life of about 7 to 10 days, for which food companies often do not specify if any conservation methods were used during processing; and (iii) street sales in coastal regions: In areas like the Grande Vitória Region in Espírito Santo State, coconut water is frequently sold from street carts on beaches. Coconuts are opened on-site, and the liquid may be poured into metallic cooling coils placed inside thermal boxes surrounded by ice, then bottled for consumption either on the beach or to take home.

While the coconut water inside the fruit is generally sterile, contamination can occur during extraction and bottling if proper hygiene practices are not followed. This can lead to the presence of high levels of spoilage and pathogenic microorganisms in the commercially available coconut water, including those from the *Enterobacteriaceae* family [3,4,5]. The *Enterobacteriaceae* comprises various commensal, pathogenic, and opportunistic bacteria. Their presence can indicate inadequate hygiene during food handling. Moreover, these bacteria are particularly concerning due to their potential for antimicrobial resistance (AMR), with some strains showing multi-drug resistance (MDR). In 2019, AMR was identified as the leading cause of deaths related to infectious diseases, predominantly affecting low- and middle-income countries [6].

Resistant *Enterobacteriaceae* strains often harbor mobile genetic elements that facilitate the spread of resistance genes, posing a significant threat to global public health [7]. Notable among these mobile genetic elements are the *bla*_CTX-M-1_, *bla*_CTX-M-2_ [8], and *bla*_CTX-M-9_ genes [9], which are associated with the production of extended-spectrum β-lactamases (ESBLs). These ESBLs confer resistance to a broad range of β-lactam antibiotics and cephalosporins. Additionally, the *bla*_CMY-1_ and *bla*_CMY-2_ genes, linked to AmpC β-lactamases, provide resistance to first-, second-, and third-generation cephalosporins [10]. Furthermore, the *bla*_KPC_ gene, responsible for *Klebsiella pneumoniae* carbapenemase, and the *bla*_NDM_ gene, associated with New Delhi metallo-β-lactamase, are primarily responsible for resistance to carbapenems [11].

This manuscript presents a cross-sectional study conducted in the Grande Vitória Region, Brazil, with the following objectives: (i) to determine whether there is a difference in contamination levels between coconut water samples sold from street carts with cooling coils on beaches and natural versions sold in bakeries; (ii) to isolate and identify the species of *Enterobacteriaceae* present in these coconut water samples; (iii) to determine the phenotypic antimicrobial resistance profile of the enterobacteria isolates; and (iv) to detect the presence of the resistance genes *bla*_CTX-M-1_, *bla*_CTX-M-2_, *bla*_CTX-M-9_, *bla*_CMY-1_, *bla*_CMY-2_, *bla*_KPC_, and *bla*_NDM_ in the isolates.

To the best of our knowledge, this is the first study aiming to evaluate the phenotypic and genotypic antimicrobial resistance profiles of enterobacteria present in coconut water samples.

## 2. Material and Methods

### 2.1. Sample Collection

**Figure 1:**
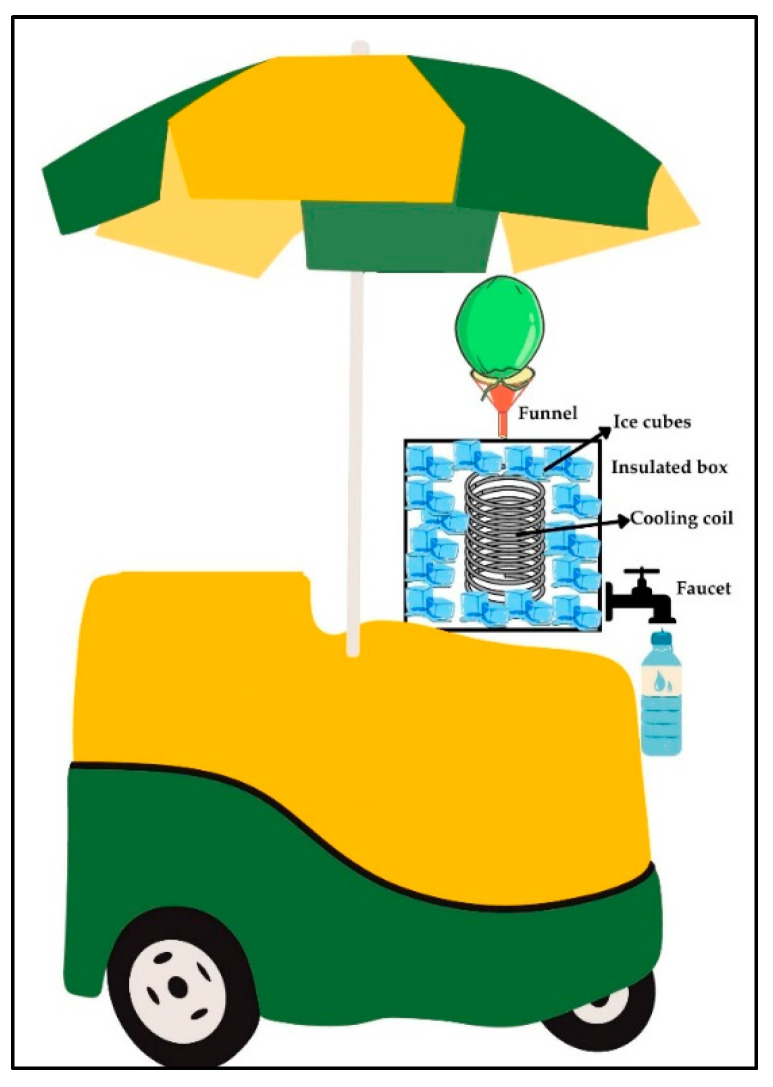
A representative drawing of a street coconut cart (street stall) selling coconut water, located on the beaches of the Grande Vitória Region, Brazil. It is noted that the cart has an insulated box with a metallic cooling coil surrounded by ice cubes inside, used to reduce the temperature of the coconut water as it passes through until it is dispensed from a faucet into cups or plastic bottles (Figure created using Canva website: https://www.canva.com accessed on 8 July 2024).

To achieve these objectives, 31 samples of coconut water (500 mL each) were collected from the Grande Vitória Region. The samples were divided into two groups: (i) Street cart samples: 16 samples were collected from six street carts (1, 2, 3, 4, 5, and 6) equipped with cooling coils (Figure 1). Specifically, two samples were obtained from street carts 1 and 4, and three samples each were taken from carts 2, 3, 5, and 6. (ii) Bakery samples: Fifteen samples were sourced from bakeries selling the “natural version” of coconut water. These samples, previously bottled and refrigerated, came from five different bakeries (1, 2, 3, 4, and 5), each associated with a distinct commercial brand. Each brand was produced by a different food company and sold in bakeries.

**Figure 2:**
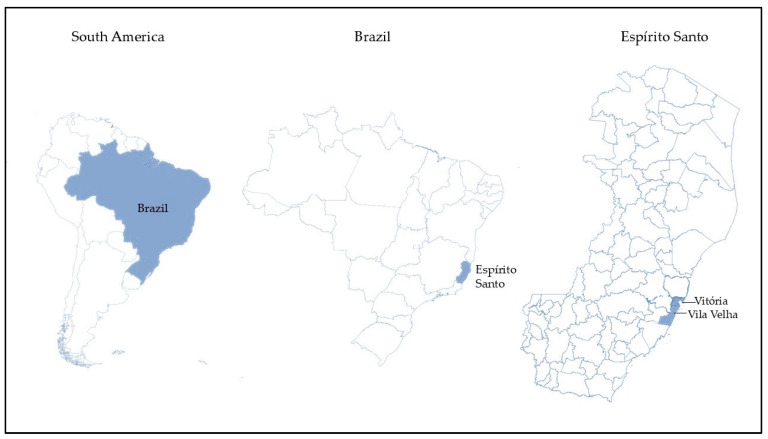
Map of the area where samples of coconut water were acquired during the year 2023 for microbiological analysis. Samples from the municipalities of Vila Velha and Vitória are included. These municipalities are part of the Vitória Region (the capital city’s macroregion), located in the state of Espírito Santo, Brazil.

For the street carts, carts labeled 1, 2, 3, 4, and 6 were in the municipality of Vila Velha, while only cart 5 was situated in the city of Vitória (Figure 2). Regarding the commercial establishments, those labeled 1, 4, and 5 were in Vitória, whereas establishments 2 and 3 were in Vila Velha. Samples from each commercial establishment or street cart were collected with a minimum interval of 15 days between collections.

### 2.2. Microbiological Analyses and Enterobacteria Identification Using MALDI-TOF

**Figure 3:**
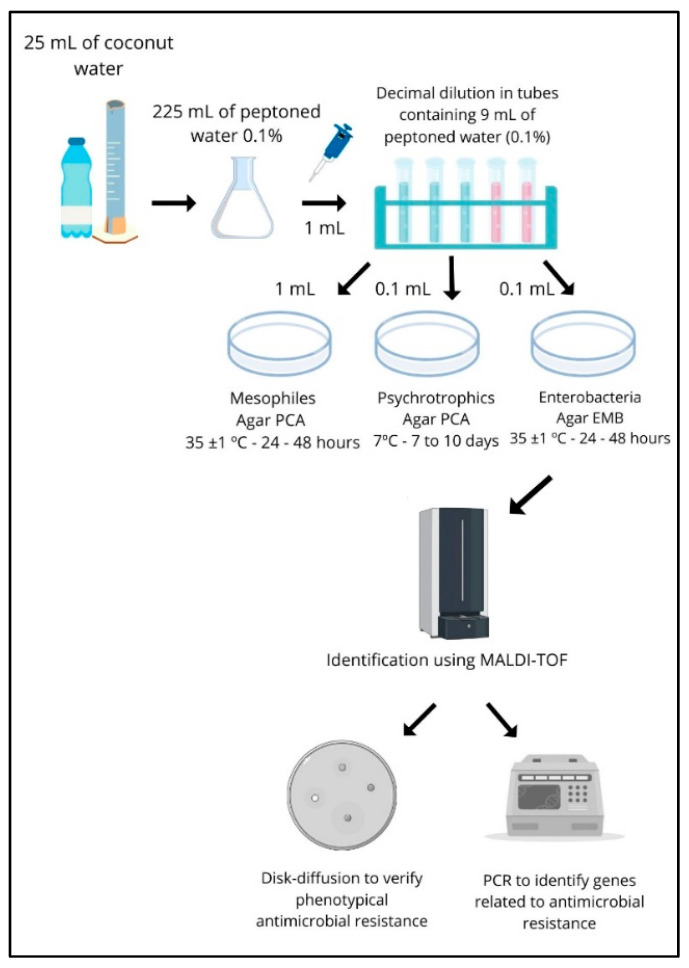
A flowchart representing the laboratory analyses conducted in the study. Briefly, the samples were subjected to serial decimal dilution. Then, they were used for inoculation in Petri dishes containing culture media to determine counts of mesophilic and psychrotrophic aerobic microorganisms and detect the presence of suggestive colonies of *Escherichia coli*. Brilliant green colonies were then identified using the MALDI-TOF and subsequently used for the evaluation of antimicrobial resistance profiles by the disk diffusion test and for PCR to verify the presence of genes related to antimicrobial resistance (figure created using Canva website: https://www.canva.com 8 July 2024).

The samples were transported in thermal boxes with recyclable ice to the laboratory and were kept refrigerated (<5 °C) until microbiological analyses were performed according to the procedure outlined in Figure 3. After the samples were received in the laboratory, the microbiological analyses were initiated within a maximum of 24 h.

A 25 mL aliquot from each sample was used to prepare serial decimal dilutions in 225 mL of sterile 0.1% peptone water (Kasvi^®^, Curitiba, Brazil), starting with a 10⁻¹ dilution and continuing with further dilutions. The study assessed the populations of mesophilic and psychrotrophic microorganisms to determine the level of contamination in the coconut water samples. Mesophilic bacteria were cultured using the pour plate technique on plate count agar (PCA) (Kasvi^®^, Curitiba, Brazil), following the APHA 08:2015 guidelines [12]. In this procedure, 1 mL of each dilution was placed into a sterile Petri dish, followed by the addition of 15 mL of liquid agar. The plates were then incubated at a temperature of 35 ± 1 °C for 24–48 h. Plates containing between 25 and 250 colony-forming units (CFU) were counted [12]. Psychrotrophic bacteria were cultured on plates prepared and incubated according to the APHA 13.61:2015 guidelines [13]. In this method, 100 µL of each dilution was applied to the surface of plates containing PCA agar and evenly spread using a Drigalsk–i loop. The plates were then incubated at 7 ± 1 °C for 7–10 days. Plates containing between 25 and 250 colony-forming units (CFU) were counted [13].

Additionally, we aimed to specifically isolate *Escherichia coli* from the samples. To do this, 0.1 mL of the 10^−1^ dilution was inoculated onto eosin methylene blue (EMB) agar (Kasvi^®^, Curitiba, Brazil) and incubated at 35 °C for 24 to 48 h. Colonies exhibiting a metallic green sheen were considered presumptive *E. coli* and were subsequently identified using matrix-assisted laser desorption/ionization–time-of-flight (MALDI-TOF) mass spectrometry [14] using the equipment Microflex LT/SH Bruker, Bruker, Billerica, MA, USA.

Following isolation, these presumptive *E. coli* isolates were subjected to antimicrobial resistance profiling using the disk diffusion test [15]. Additionally, PCR reactions were performed to detect genes associated with extended-spectrum β-lactamases (ESBLs), AmpC β-lactamases, *Klebsiella pneumoniae* carbapenemase (KPC), and New Delhi metallo-β-lactamase (NDM).

### 2.3. Disk-Diffusion Test

The disk-diffusion test was used to evaluate the phenotypic antimicrobial resistance profile of the isolates [15]. The isolates were prepared by inoculating them onto nutrient agar (Kasvi^®^, Curitiba, Brazil) and incubating at 37 °C for 24 h to prepare the inoculum for the disk-diffusion test. The inoculum was prepared in tubes containing 3 mL of 0.85% saline solution, with the solution being turbidified with the culture until reaching an OD625 nm of 0.1 to 0.2 (0.5 scale MacFarland’s standard). Subsequently, these inoculums were streaked onto plates containing Mueller–Hinton Agar (Kasvi^®^, Curitiba, Brazil) using a sterile swab, and disks containing selected antimicrobial agents were placed on the plates. Nine antimicrobial groups were used:

(1) β-Lactams–amoxicillin + clavulanic acid (30 µg) (AMC), ampicillin (10 µg) (AMP), imipenem (10 µg) (IPM), and penicillin (10 UI) (PEN);
(2) Cephalosporins–cefalexin (30 µg) (CFE) and ceftriaxone (30 µg) (CRO);
(3) Aminoglycoside–streptomycin (10 µg) (EST) and gentamicin (10 µg) (GEN);
(4) Tetracyclines–tetracycline (30 µg) (TET) and doxycycline (30 µg) (DOX);
(5) Quinolones–ciprofloxacin (5 µg) (CIP) and ofloxacin (5 µg) (OFX);
(6) Sulfonamides–sulfamethoxazole + trimethoprim (25 µg) (SUT);
(7) Amphenicol–chloramphenicol (30 µg) (CLO);
(8) Nitrofurans–nitrofurantoin (300 µg) (NIT);
(9) Phosphonic acids–fosfomycin (200 µg) (FOS).

The antimicrobial disks CIP, TET, AMP, FOS, EST, NIT, and IPM were provided by *Diagnósticos Microbiológicos Especializados* (DME) (Araçatuba, São Paulo, Brazil), while the disks CFE, SUT, PEN, OFX, AMC, CRO, DOX, CLO, and GEN were provided by the brand *Centro de Controle e Produtos para Diagnóstico* LTDA (CECON) (São Paulo, São Paulo, Brazil).

The plates were incubated for 24 h at 37 °C. After incubation, the diameters of the inhibition zones were measured and classified as susceptible, resistant, or intermediate sensitivity based on the parameters set by the Clinical & Laboratory Standards Institute 2023 [16]. Quality control strains were not used in this analysis. An isolate was classified as multi-drug resistant (MDR) if it exhibited resistance to at least three different antibiotic groups.

### 2.4. Detection of Antimicrobial Resistance Genes

Bacterial DNA extraction for resistance gene analysis was performed using the boiling method. Colonies were incubated in brain heart infusion broth (BHI) (Kasvi^®^, Curitiba, Brazil) at 37 °C for 24 h. Subsequently, 0.1 mL of the culture was subjected to boiling at 100 °C for 10 min [17]. The DNA samples were quantified using a NanoDropOne spectrophotometer (Thermo Scientific, Waltham, MA, USA).

PCR assays were conducted on a thermocycler (Eppendorf, Mastercycler Nexus GSX1, Hamburg, Germany) to detect the genes *bla*_CTX-M-1_, *bla*_CTX-M-2_ [8], *bla*_CTX-M-9_ [9], *bla*_CMY-1_, *bla*_CMY-2_ [10], *bla*_KPC_, and *bla*_NDM_ [11]. A mix containing 0.4 μL of 10 mM dNTPs, 2 μL of 10X Taq polymerase buffer, 0.8 μL of 50 mM MgCl2, 1 μL of 5 pmol primer, 0.2 μL of Taq polymerase, 1 μL of DNA template, and ultrapure water was used to reach a total volume of 20 μL. The information regarding the primers used is available in Table 1. After each PCR, 5 μL of loading dye (0.25% bromophenol blue in 50% glycerol) was added to the reaction product, and a 100 bp DNA ladder (Invitrogen, Waltham, MA, USA) was applied to a 1.5% agarose gel containing ethidium bromide (1 μg/mL) in Tris-Borate-EDTA (TBE) buffer. The samples were separated by electrophoresis (70 V/1 h). The amplification products were visualized by exposing the gel to UV light on a transilluminator (Bio-Rad, GelDoc ^TM^ XR+, Hercules, CA, USA).

### 2.5. Statistical Analyses

Statistical analysis was performed on the counts of mesophilic and psychrotrophic microorganisms from the two sample groups. Data normality was assessed using the Shapiro–Wilk test to compare coconut water samples from street carts and bakeries based on microorganism counts. Due to the lack of normality, the data were log-transformed using a base-10 logarithm with the transformation log (x + 1) to achieve a normal distribution. A *t*-test was then used to compare the mean counts, with a significance level set at 5%. All analyses were conducted using the R software, version 4.3.3. The graphs were created using the ggplot2 package in R.

## 3. Results and Discussion

The results of the counts of mesophilic and psychrotrophic aerobic microorganisms are presented in Table 2.

The mesophilic and psychrotrophic microorganism counts in the analyzed samples from both product groups were generally high, indicating poor overall microbiological quality. This is likely due to inadequate hygienic practices during extraction, production, and commercialization, as similarly reported in other regions of Brazil [18,19] and within the same study area [5]. Educational initiatives that emphasize proper food handling and manufacturing practices, particularly focusing on the sanitization of equipment and utensils, could help improve the quality of coconut water sold in the region [3,20]. However, it is important to note that there are no established legal limits for these microorganism groups in this type of food in Brazil.

**Figure 4:**
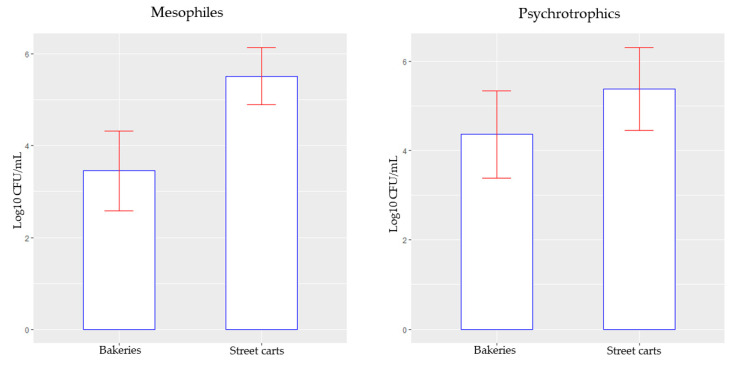
Bar chart of the mean values and standard deviation of mesophilic and psychrotrophic microorganism counts (log10 CFU/mL) in the two groups of coconut water samples analyzed (from bakeries and street carts) in this study.

The *t*-test revealed a statistically significant difference (*p* = 0.0002462) between the mean counts of mesophilic aerobic microorganisms in samples from street carts with cooling coils (5.515 log10 CFU/mL) compared to those from bakeries (3.453 log10 CFU/mL). In contrast, there was no significant difference (*p* = 0.1198) in the mean counts of psychrotrophic microorganisms between street carts (5.373 log10 CFU/mL) and bakeries (4.362 log10 CFU/mL) (Figure 4).

The higher average counts of mesophilic microorganisms in street cart samples suggest deficiencies in temperature and sanitation control during coconut water processing. A study conducted in Brazil that evaluated the hygienic practices of street carts found that the cleaning of carts and cooling coils generally follows a schedule established by the street vendor according to their own perception, which varies from every two days to once a month. The cart is usually cleaned with detergent and water, but some vendors only use water. However, the cooling coil is typically sanitized using only water and, in a frequency lower than that of the carts, often only once a month, as reported by some vendors. Additionally, these establishments commonly exhibit inadequate practices such as wearing adornments on the hands, long fingernails, the absence of gloves and hairnets, and the lack of facilities for hand hygiene and water supply, and these practices are known to increase food contamination [3].

Although there was no statistically significant difference in the average counts of psychrotrophic microorganisms, it was noted that the average was higher in coconut water samples from street carts. It is important to consider that the street cart samples were extracted from the fruit and bottled at the time of purchase and then analyzed within a maximum of 24 h. In contrast, the bakery samples had been refrigerated for several days, as indicated by the shelf-life on the label, which averaged 7 to 10 days. This prolonged refrigeration allowed for the multiplication of psychrotrophic microorganisms, which can thrive at these temperature ranges [13]. Therefore, the samples from street carts, which were obtained and analyzed in a short period, likely exhibited poorer microbiological quality in terms of psychrotrophic microorganism counts due to inadequate hygienic practices and insufficient sanitation of the cooling coils [3].

In this study, 13 isolates that exhibited a metallic green sheen on EMB agar were obtained and subsequently identified using MALDI-TOF. They were obtained mainly from street carts (nine isolates from street carts and four from bakeries). Surprisingly, none of these isolates were identified as *E. coli*; instead, they were identified as *Citrobacter freundii* (one isolate), *Enterobacter bugandensis* (three isolates), *Enterobacter kobei* (one isolate), *Enterobacter roggenkampii* (one isolate), *Klebsiella pneumoniae* (two isolates), and *Kluyvera ascorbata* (five isolates) (Table 3). All these genera of *Enterobacteriaceae* have been previously reported as potential false positives for *E. coli* on EMB agar, as they can also produce colonies with a metallic green sheen [21]. *K. pneumoniae*, *Citrobacter* spp., and *Enterobacter* spp. resistant to third-generation cephalosporins as well as carbapenem-resistant *K. pneumoniae* and *Enterobacter* spp. are classified as critical priority pathogens on the WHO’s 2024 bacterial priority list [22]. Therefore, the antimicrobial resistance profile of our isolates is crucial for understanding the occurrence of AMR in the studied area.

Among the isolates, three (*E. bugandensis*, *K. pneumoniae*, and *K. ascorbata*) were classified as multidrug-resistant (MDR); however, none of the isolates in this study exhibited resistance to carbapenems or ceftriaxone. The *bla*_CTX-M-2_ gene was detected in eight isolates (*C. freundii* (one isolate), *E. bugandensis* (one), *E. kobei* (one), and *K. ascorbata* (five isolates)) (Table 3). The other resistance genes *bla*_CTX-M-1_, *bla*_CTX-M_-_9_, *bla*_CMY-1_, *bla*_CMY-2_, *bla*_KPC_, and *bla*_NDM_ were not detected in any of the isolates.

The single isolate of *Citrobacter freundii* exhibited the presence of the *bla*_CTX-M-2_ gene and demonstrated phenotypic resistance to cephalexin and ofloxacin. Although *C. freundii* is a commensal bacterium typically found in the gastrointestinal tracts of humans and animals, it is also recognized as an opportunistic pathogen capable of causing foodborne outbreaks [23,24] and other infections in humans [25]. This microorganism is known to harbor extended-spectrum β-lactamase (ESBL) and plasmid-mediated quinolone-resistance determinants [25,26]. In this study, resistance to both quinolones (ofloxacin) and cephalosporins (cephalexin) was observed, aligning with previous reports of such resistance in the *Citrobacter* genus [25,26]. Although the *bla*_CTX-M-9_gene—previously reported in *Citrobacter* isolates from humans, foods, and environmental sources [25,26]—was not detected, the presence of the *bla*_CTX-M-2_ gene was confirmed. The widespread dissemination of CTX-M-type ESBLs is significantly altering the epidemiology of these enzymes, making them the most common ESBLs found in *Enterobacteriaceae*, which confer resistance to third-generation cephalosporins [27]. This isolate was susceptible to ceftriaxone, a third-generation cephalosporin, but exhibited resistance to cephalexin, a first-generation cephalosporin. It is known that enterobacteria can develop resistance to cephalexin through mechanisms other than ESBL-mediated inactivation, such as the disruption or downregulation of OmpF porins [28].

In this study, five isolates from the genus *Enterobacter* were obtained, including three *Enterobacter bugandensis* (one of which was classified as MDR), one *Enterobacter kobei*, and one *Enterobacter roggenkampii*. These three species of *Enterobacter* have previously been implicated in bloodstream infections [29]. Notably, *E. bugandensis*, which has recently emerged as a highly pathogenic species within this genus [30], has been associated with outbreaks in neonates due to its ability to colonize incubators [31]. In our study, the *bla*_CTX-M-2_ gene was detected in both *E. kobei* and *E. bugandensis*. As shown in Table 3, several isolates from the genus *Enterobacter* exhibited resistance to cephalosporins (cephalexin) and β-lactams (amoxicillin + clavulanic acid and ampicillin). One *E. bugandensis* isolate, which carried the *bla*_CTX-M-2_ gene, was classified as MDR due to its simultaneous resistance to β-lactams (AMC, AMP, and PEN), cephalosporins (CFE), and phosphonic acids (FOS). The presence of the *fosA2* gene, which is associated with fosfomycin resistance, was previously reported in an *E. bugandensis* (ST 921) isolate from a vegetable in the USA that also showed resistance to carbapenems [32]. Phenotypic resistance to β-lactams is well documented in this bacterial genus, as also observed in our study. Additionally, *bla*_KPC_ and *bla*_CTX-M-1_ genes have been identified in *E. bugandensis* isolates from water and wastewater samples [33,34], and *bla*_NDM_-*5* was detected in an isolate from cattle vermicompost [35]. However, in our study, none of the isolates carried these genes; only *bla*_CTX-M-2_ was detected. The absence of *bla*_KPC_ and *bla*_NDM_ genes aligns with the observed sensitivity to carbapenems in the isolates analyzed in this study.

Two *Klebsiella pneumoniae* isolates were obtained from the samples, with one classified as multidrug-resistant (MDR) but lacking the *bla*_CTX-M-2_ gene. This MDR isolate demonstrated resistance to β-lactams (AMP and PEN), cephalosporins (CFE), and nitrofurans (NIT). *K. pneumoniae* is an opportunistic pathogen capable of causing severe organ damage and life-threatening conditions, particularly in individuals with compromised immune systems, existing comorbidities, or disrupted physical barriers [36,37]. This microorganism is known to cause various infections, including urinary tract infections, cystitis, pneumonia, surgical wound infections, endocarditis, septicemia, pyogenic liver abscesses, and endogenous endophthalmitis. The global rise in antimicrobial resistance (AMR), including the emergence of MDR isolates, is of growing concern [38]. The presence of carbapenemase-producing strains of *K. pneumoniae* is considered a significant global threat [39]; however, this was not observed in our study.

Notably, the MDR *K. pneumoniae* isolate in our study exhibited resistance to nitrofurantoin. Although nitrofurantoin is commonly used to treat infections caused by MDR *K. pneumoniae*, resistance to this drug has been documented and linked to the efflux pump regulator *oqxR* [40]. Nitrofurantoin resistance may indicate the presence of an extensively drug-resistant (XDR) phenotype within *Enterobacteriaceae*, which carries multiple AMR and efflux pump genes [41]. Similar to the other bacterial genera detected in this study, the *K. pneumoniae* isolate also showed resistance to β-lactams (AMP and PEN) and cephalosporins (cephalexin), a pattern frequently observed in *K. pneumoniae* isolates from hospital settings [42]. Although the *bla*_CTX-M-2_ gene has been reported in *K. pneumoniae* strains associated with hospitals and intensive care units samples in Brazil [43,44,45], it was not detected in either of the two isolates obtained in our study.

In this study, five *Kluyvera ascorbata* isolates were obtained, all of which harbored the *bla*_CTX-M-2_ gene, with one isolate classified as multidrug-resistant (MDR). The MDR isolate exhibited resistance to β-lactams (AMC, AMP, and PEN), cephalosporins (CFE), and phosphonic acids (FOS). *K. ascorbata* is recognized as an opportunistic pathogen with a rising incidence [46] and has been implicated in septic shock [47,48], urinary tract infections [47,49], and biliary tract infections [50]. Interestingly, *K. ascorbata* has been proposed as the progenitor of the plasmid-encoded CTX-M-2 subgroup, as the earliest chromosomal variants reported in this species exhibited a very high amino acid identity with CTX-M-2 [51]. This likely explains why the *bla*_CTX-M-2_ gene was identified in all the isolates in this study. Additionally, the presence of a *fosA* gene related to fosfomycin resistance, identical to that found in *Escherichia coli*, was reported in a *K. ascorbata* isolate from hospital sewage [52], supporting our findings regarding fosfomycin resistance in the MDR isolate from our study.

It is important to highlight that none of the isolates in this study carried the *bla*_NDM_, *bla*_KPC_, *bla*_CMY-1_, or *bla*_CMY-2_ genes. The New Delhi metallo-β-lactamase (NDM) enzyme, encoded by the *bla*_NDM_ gene, can hydrolyze nearly all β-lactam antibiotics, including newer agents like ceftazidime–avibactam. This gene has been identified in various *Enterobacteriaceae* species on plasmids [53], which were possibly absent in the isolates obtained in this study. A study evaluating 360 samples of vegetables and salad also found no presence of the *bla*_NDM_ gene in any of the *Enterobacteriaceae* isolates [54]. Carbapenem resistance can also be attributed to the presence of the *bla*_KPC_ gene, typically located on plasmids. This gene was identified in *Citrobacter*, *Enterobacter*, and *Klebsiella pneumoniae* isolates from a Portuguese river [55]. However, studies indicate that the prevalence of the *bla*_KPC_ gene is lower in isolates from tertiary-care hospitals and vegetables compared to other carbapenem-resistance genes [56,57]. Although the *bla*_CMY-1_ and *bla*_CMY-2_ genes, associated with AmpC β-lactamase production, were not detected in the isolates from this study, *bla*_CMY-1_ was reported in *K. pneumoniae* from elderly patients in Tehran, Iran [58]. However, these genes are more commonly described in other enterobacteria such as *Escherichia*, *Salmonella*, and *Klebsiella* species.

Given the findings of this study and the widespread consumption of coconut water in the sampled region, without any microbial inactivation treatment, this product poses a potential health risk. Coconut water is consumed by a broad range of individuals, including children, adults, and the elderly, due to its reputation as a healthy and hydrating beverage [1,2]. However, the opportunistic pathogens detected in this study, particularly the multidrug-resistant (MDR) strains, could colonize the intestines of consumers. This colonization may facilitate the transfer of mobile genetic elements to other *Enterobacteriaceae* in the gut [59], contributing to the spread of antimicrobial resistance (AMR) in the region. Additionally, these opportunistic pathogens could potentially cause infections that are difficult to treat [60]. Therefore, it is crucial to adopt better hygienic food-handling practices, particularly the sanitization of equipment and utensils that come into direct contact with coconut water [3,61], to prevent the spread of AMR in this area.

It is important to acknowledge some limitations of this study: (1) A small number of coconut water samples were analyzed, yet this sample size was sufficient to detect statistically significant differences between the groups. (2) The study may not have captured the full spectrum of *Enterobacteriaceae* present in the samples due to the exclusive use of EMB agar and the focus on identifying metallic green colonies, as the initial aim was to isolate *E. coli* rather than other enterobacteria. (3) PCR analysis was not conducted for all possible genes related to the nine different classes of antimicrobials examined in the study.

In conclusion, the coconut water sold in the Grande Vitória Region shows poor overall microbiological quality, highlighted by high counts of mesophilic and psychrotrophic microorganisms. This issue is particularly severe in samples from street carts with cooling coils. Additionally, opportunistic pathogens from the *Enterobacteriaceae* family were found to be multi-drug resistant (MDR), displaying high resistance to β-lactams and cephalosporins and possessing the bla_CTX-M-2_ gene associated with extended-spectrum β-lactamase (ESBL) production. These findings underscore the need for improved good manufacturing practices in the region to mitigate the spread of antimicrobial resistance (AMR). To our knowledge, this is the first study to document the presence of potentially pathogenic enterobacteria in coconut water samples and their associated phenotypic and genotypic profiles of AMR.

## Figures and Tables

**Table 1:** Genes, primers (forward and reverse), fragment size, annealing temperature, and references used for performing PCR to detect genes related to the phenotypic characterization of antimicrobial resistance in Enterobacteriaceae isolates identified in coconut water samples.

Gene	Primer’s Sequence (5′—3′)	Fragment Size (pb)	Annealing Temperature	Reference
*bla*_CTX-M-1_	F-TTAGGAARTGTGCCGCTGYA	688	60 °C	[8]
	R-CGATATCGTTGGTGGTRCCAT			
*bla*_CTX-M-2_	F-CGTTAACGGCACGATGAC	404	60 °C	[8]
	R-CGATATCGTTGGTGGTRCCAT			
*bla*_CTX-M-9_	F-ATGGTGACAAAGAGAGTGCA	870	55 °C	[9]
	R- CCCTTCGGCGATGATTCTC			
*bla*_CMY-1_	F-ATGCAACAACGACAATCCATCCTG	1560	60 °C	[10]
	R-TCAACCGGCCAACTGCGCCAGGAT			
*bla*_CMY-2_	F-ATGATGAAAAAATCGTTATGCT	3202	60 °C	[10]
	R-TTATTGCAGCTTTTCAAGAATGCG			
*bla*_KPC_	F-CGTCTAGTTCTGCTGTCTTG	798	52 °C	[11]
	R-CTTGTCATCCTTGTTAGGCG			
*bla*_NDM_	F-GGTTTGGCGATCTGGTTTTC	621	52 °C	[11]
	R-CGGAATGGCTCATCACGATC			

**Table 2:** Results of the mesophilic and psychrotrophic aerobic microorganisms count (log10 CFU/mL) in coconut water samples sold at street carts and bakeries in the Grande Vitória Region, Espírito Santo, Brazil.

Street Carts	Sample	Mesophiles (log10 CFU/mL)	Psychrotrophics (log10 CFU/mL)	Bakeries	Sample	Mesophiles (log10 CFU/mL)	Psychrotrophics (log10 CFU/mL)
1	1	6.3424229	6.6812413	1	1	4.0086427	5.6020611
	2	5.9444832	6.9190781		2	2.3242825	4.1461591
2	1	4.8228282	4.1761202		3	5.1335421	5.2944684
	2	5.4623995	6.0000004	2	1	3.7160869	3.5798979
	3	4.1761202	6.4149735		2	4.9444876	4.033464
3	1	6.819544	6.9731279		3	2.4487063	3.2555137
	2	7.2430381	7.8920946	3	1	2.2810334	3.3803922
	3	6.6901962	6.9867718		2	1.7853298	6.8864908
4	1	5.8260755	6.1643532		3	1.4913617	4.2304745
	2	5.3617297	4.9684876	4	1	2.363612	-
5	1	3.7076553	3.4315246		2	2.5921768	4.0374663
	2	7.3096302	2.3031961		3	2.3996737	3.0004341
	3	5.6812421	6.3074963	5	1	4.0414322	4.6627673
6	1	4.2068529	2.8000294		2	6.6989701	6.9731279
	2	4.6532222	4.9030954		3	5.5682029	6.3502482
	3	4.0000434	3.0417873				

**Table 3:** Identification results (MALDI-TOF) of species from isolates with a metallic green sheen on EMB agar, including the establishment of the sample origin. Additionally, the occurrence of the *bla*_CTX-M-2_ gene, classification as MDR, and the antimicrobial resistance profile for nine groups of antibiotics (16 antibiotics) are also presented.

Origin	Specie	*bla*_CTX-M-2_	MDR	β-lactams				Cephalosporins		Aminoglycoside		Tetracyclines		Quinolones		Sulfonamides	Amphenicol	Nitrofurans	Phosphonic acids
				AMC	AMP	IPM	PEN	CFE	CRO	EST	GEN	DOX	TET	CIP	OFX	SUT	CLO	NIT	FOS
Bakery 1	*Citrobacter freundii*	+	−	S	S	S	I	R	S	S	S	S	S	S	R	S	S	S	S
Bakery 2	*Enterobacter kobei*	+	−	R	R	S	R	R	S	S	S	S	S	S	S	S	S	S	S
Street cart 3	*Enterobacter bugandensis*	−	−	R	R	S	R	R	S	S	S	S	S	S	S	S	I	S	I
Street cart 6	*Kluyvera ascorbata*	+	−	S	S	S	R	S	S	S	S	S	S	S	S	S	S	I	S
Bakery 2	*Kluyvera ascorbata*	+	+	R	R	S	R	R	S	S	S	S	S	S	S	S	S	S	R
Bakery 5	*Kluyvera ascorbata*	+	−	S	R	S	R	I	I	S	S	S	S	S	S	S	S	S	S
Street cart 3	*Kluyvera ascorbata*	+	−	S	R	S	R	I	S	S	S	S	S	S	S	S	S	S	S
Street cart 6	*Enterobacter roggenkampii*	−	−	R	S	S	R	R	S	S	S	S	S	S	S	S	S	S	S
Street cart 6	*Enterobacter bugandensis*	−	−	R	S	S	R	R	S	S	S	S	S	S	S	S	S	S	I
Street cart 2	*Klebsiella pneumoniae*	−	−	S	R	S	R	I	S	S	S	S	S	S	S	S	S	S	S
Street cart 3	*Enterobacter bugandensis*	+	+	R	R	S	R	R	S	S	S	S	S	S	S	S	S	I	R
Street cart 4	*Kluyvera ascorbata*	+	−	S	R	S	R	R	S	S	S	S	S	S	S	S	S	S	S
Street cart 6	*Klebsiella pneumoniae*	−	+	S	R	S	R	R	S	S	I	S	S	I	S	S	S	R	S

LEGEND: + Positive and − Negative; S—Sensible, I—Intermediate, and R—Resistant; AMC—Amoxicillin + Clavulanic Acid, AMP—Ampicillin, CFE—Cefalexin, CIP—Ciprofloxacin, CLO—Chloramphenicol, CRO—Ceftriaxone, DOX—Doxycycline, EST—Streptomycin, FOS—Fosfomycin, GEN—Gentamicin, IPM—Imipenem, NIT—Nitrofurantoin, OFX—Ofloxacin, PEN—Penicillin, SUT—Sulfamethoxazole + Trimethoprim, and TET—Tetracycline. Yellow: Sensible isolates, Orange: intermediate, Red: resistant.

## Data Availability

The full data are available in this manuscript.
